# Short- and Long-Term Effect of Parkinson’s Disease Multimodal Complex Treatment

**DOI:** 10.3390/brainsci11111460

**Published:** 2021-11-03

**Authors:** Konstantin G. Heimrich, Tino Prell

**Affiliations:** 1 Department of Neurology, Jena University Hospital, 07747 Jena, Germany; 2 Department of Geriatrics, Halle University Hospital, 06120 Halle, Germany; tino.prell@uk-halle.de

**Keywords:** Parkinson’s disease, multidisciplinary care, health services research, depression, quality of life

## Abstract

Parkinson´s disease multimodal complex treatment (PD-MCT) is a multidisciplinary inpatient treatment option increasingly applied in Germany. However, data on its effectiveness are rare. Data were collected at the Department of Neurology of the University Hospital Jena, Germany. In 2019, 159 patients were admitted to our neurology ward for PD-MCT. Patients were followed for up to 12 months, and their data were retrospectively analyzed to assess the short- and long-term treatment effects. The treatment led to an improvement in motor function assessed by Movement Disorder Society sponsored revision of the unified Parkinson´s disease rating scale part III (MDS-UPDRS III) and motor performance (Tinetti test). Improvement of MDS-UPDRS III was associated with lower age, higher MDS-UPDRS III at admission, and less depression (assessed by Hospital Anxiety and Depression Scale and Beck-Depression Inventory II). One month after the hospital stay, 36.8% of the patients reported feeling better, while 32.6% reported feeling worse. If the patients were not depressed, they were more likely to have reported feeling better. PD-MCT is an effective inpatient treatment option. However, to improve patients’ satisfaction, screening and treatment for depression is essential. The effectiveness of different treatment durations has to be elucidated in further studies.

## 1. Introduction

Parkinson’s disease is one of the most common neurodegenerative disorders. It is characterized by motor symptoms and a plethora of nonmotor symptoms [1]. PD is a progressive disorder leading to severe disability [2]. During the disease course and with worsening motor and nonmotor function, individual medical treatment needs to be continuously adapted. Because of the wide range of symptoms, multidisciplinary treatment approaches are essential to maintain and improve patient health. For this purpose, in Germany, a multidisciplinary PD inpatient treatment concept called Parkinson’s disease multimodal complex treatment (PD-MCT) is increasingly being applied [3]. In addition to pharmacological adjustments, PD-MCT includes interprofessional treatment by physiotherapists, occupational therapists, speech and language therapists, and psychologists. Thereby, psychologists perform neuropsychological tests, among other activities, to reveal depressive symptoms assessed by, e.g., Hospital Anxiety and Depression Scale (HADS-D) or Beck-Depression Inventory II (BDI-II).

PD-MCT is an integrated part of the German health insurance system and takes place in accordance with the requirements of the Operation and Procedure Classification System (OPS) as an official coding system for medical procedures. Therefore, detailed requirements exist for PD-MCT. A minimum of 7.5 h of treatment per week and weekly team meetings with documentation of previous treatment results and further treatment goals are required. The duration of PD-MCT varies from a minimum of 7 days to more than 21 days. One can distinguish between PD-MCT over a period of 7–13 days (OPS8-97d.0), 14–20 days (OPS8-97d.1), and for at least 21 days (OPS8-97d.2). In Germany, the majority of patients are treated for at least 14 days (OPS8-97d.1) [3]. The longer treatments (OPS8-97d.1 and OPS8-97d.2) have a positive effect on motor and nonmotor symptoms, as shown in two smaller nonrandomized studies with 126 and 47 PD patients [4,5,6]. Only one of the two studies also analyzed whether this positive effect persisted after hospital discharge in a follow-up examination of 47 patients after six weeks [4,5]. Data regarding the effectiveness of the shorter OPS8-97d.0 (7–13 days) are missing, as well as data regarding the long-term effect of PD-MCT. With this study, we aimed to provide additional data about PD-MCT to identify predictors of motor improvement. We also describe the long-term dynamics of health-related quality of life (HR-QoL) after PD-MCT.

## 2. Materials and Methods

Data on all patients treated in the PD-MCT from 1 January 2019 until 31 December 2019 at the Department of Neurology of the University Hospital Jena, Germany were collected (*n* = 159). This included the PD-MCTs for 7–13 days (OPS8-97d.0), 14–20 days (OPS8-97d.1), and for at least 21 days (OPS8-97d.2) and patients with idiopathic PD and atypical Parkinsonian syndromes (APS). The following baseline variables were collected: sociodemographic parameters, Hoehn and Yahr stage, Movement Disorder Society sponsored revision of the unified Parkinson´s disease rating scale (MDS-UPDRS) Part I–IV, Tinetti test, levodopa equivalent daily dose (LEDD), nonmotor symptoms questionnaire (NMSQ), measures of depressive symptoms (HADS-D and BDI-II), Montreal Cognitive Assessment (MoCA), and health-related QoL assessed with the Short-Form 12 (SF-12). Because of the structural changes in our neuropsychology section for the initial patients, the HADS-D (*n* = 118) was used, and for subsequent patients, the BDI-II (*n* = 118). Patients were classified as depressed if the HADS-D ≥ 8 [7] and/or the BDI-II ≥ 14 [8].

To assess the short-term effect of PD-MCT, at discharge the following variables were collected again: MDS-UPDRS III, Tinetti test, and LEDD. In addition, one month after the patients were discharged from the treatment, a follow-up was conducted to assess a persistent short-term effect of PD-MCT, and patients were called and asked if they felt better, about the same or worse in comparison to the time of discharge from the hospital.

One year after PD-MCT, the SF-12 was assessed by telephone (three attempts were made to reach the patients) to assess the long-term effect of PD-MCT.

## 3. Results

### 3.1. Baseline Characteristics

**Figure 1:**
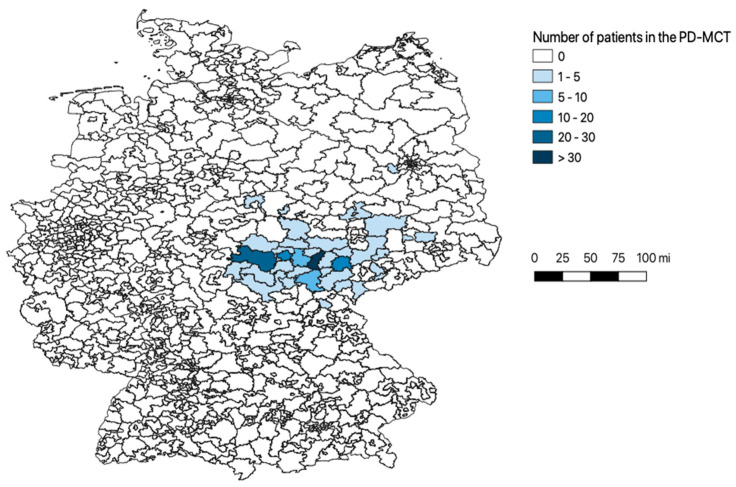
Study population, catchment area (zip code). The figure shows a map of Germany divided by zip code as the catchment area; the color coding represents the number of patients treated in the PD-MCT at our neurology ward in 2019. PD-MCT: Parkinson’s disease multimodal complex treatment.

On average, patients traveled 53.6 ± 71.3 miles to attend the PD-MCT. Figure 1 shows the catchment area by zip code region. The demographic and clinical characteristics of the patients are shown in Table 1.

Of 159 patients, 134 (84.3%) had PD, and 25 (15.7%) had APS (multiple system atrophy: *n* = 8; cortico-basal degeneration: *n* = 7; progressive supranuclear palsy: *n* = 6; dementia with Lewy bodies: *n* = 4). The majority of patients were male, aged above 60 years, and presented with postural instability (Hoehn and Yahr ≥ 3). The MDS-UPDRS III indicated moderate to severe motor impairment. Motor complications according to the MDS-UPDRS IV were present in 137 patients (86.2%). The majority (152, 95.6%) received dopaminergic medication at the time of admission; three patients with PD and four patients with APS did not have dopaminergic medication at the time of admission to the hospital. According to the NMSQ, patients reported on average 11.6 ± 5.1 NMS. Eighty-two patients (51.6%) were identified as depressed. According to the MoCA, 8.8% had normal cognition, 38.4% mild cognitive impairment, and 49.7% PD dementia (MoCA range 6 to 29).

### 3.2. Short-Term Effect of Multimodal Complex Treatment

**Figure 2:**
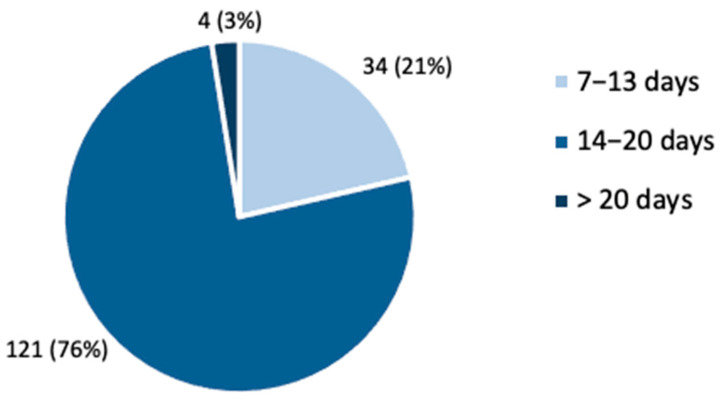
Study population (*n*, %) according to the duration of PD-MCT.

**Figure 3:**
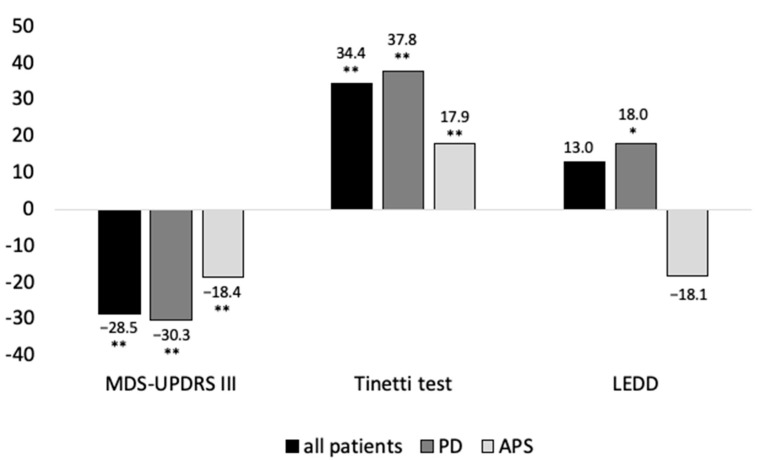
Relative changes (%) in clinical outcome parameters from baseline to discharge. *: *p* < 0.05; **: *p* < 0.005.

Most patients (121, 76%) were treated for 14–20 days (OPS8-97d.1), 34 patients (21%) in the shorter OPS8-97d.0 and four patients (3%) in the longer OPS8-97d.2 (see Figure 2). Independent t-tests revealed no differences between patients treated for 7–13 days and patients treated for at least 14 days regarding disease severity assessed by MDS-UPDRS III at admission (*t* = −0.59; *p* = 0.556). From admission to discharge from hospital, MDS-UPDRS III improved by 9.6 ± 8.0 points (*p* < 0.001), and functional motor performance (Tinetti test) improved by 2.7 ± 3.8 points (*p* < 0.001). Three patients showed worsening of MDS-UPDRS III. The mean LEDD increased from 869.6 ± 515.4 to 934.6 ± 522.5 mg (*p* = 0.035) in PD and decreased from 407.0 ± 374.0 mg to 295.4 ± 285.9 mg (*p* = 0.071) in APS. Figure 3 summarizes the relative changes in the clinical parameters after PD-MCT. After PD-MCT, the MDS-UPDRS III improved (lower values) by 30.3% in patients with PD and 18.4% in patients with APS. Likewise, Tinetti test improved (higher values) by 37.8% in patients with PD and 17.9% in patients with APS. Accordingly, motor improvement was more pronounced in patients with PD than in those with APS.

The absolute changes of MDS-UPDRS III (t = −0.72; *p* = 0.473), Tinetti test (*t* = −0.74; *p* = 0.464) and LEDD (*t* = −0.17; *p* = 0.869) after PD-MCT did not differ between the shorter (OPS8-97d.0) and the longer (OPS8-97d.1) PD-MCT in the entire cohort. Considering only patients with idiopathic PD (without APS), the changes in MDS-UPDRS III (*t* = −1.1; *p* = 0.277), Tinetti test (*t* = 0.24; *p* = 0.633), and LEDD (*t* = −0.4; *p* = 0.720) did not differ between the shorter OPS8-97d.0 and longer OPS8-97d.1 groups.

The improvement of MDS-UPDRS III for the entire cohort (PD and APS) after PD-MCT correlated positively with MDS-UPDRS III at admission (*r* = 0.34; *p* < 0.001) and negatively with depression (*r_s_* = −0.19; *p* = 0.022) but not with age (*p* = 0.281), sex (*p* = 0.089), LEDD (*p* = 0.173), Hoehn & Yahr stage (*p* = 0.192), disease duration (*p* = 0.222), NMSQ (*p* = 0.724), or MoCA (*p* = 0.113). For the PD-only cohort (without APS), improvement in the MDS-UPDRS III after PD-MCT correlated positively with MDS-UPDRS III at admission (*r* = 0.37; *p* < 0.001) and negatively with depression (*r_s_* = −0.23; *p* = 0.011) but not with age (*p* = 0.176), sex (*p* = 0.512), LEDD (*p* = 0.632), Hoehn & Yahr stage (*p* = 0.102), disease duration (*p* = 0.852), NMSQ (*p* = 0.631), or MoCA (*p* = 0.081).

In the linear regression, improvement of the MDS-UPDRS III scores during PD-MCT was associated with lower age (beta = −0.22; *p* = 0.005), higher MDS-UPDRS III scores at admission (beta = 0.60; *p* < 0.001), and less depression (beta = −0.19; *p* = 0.016) (F(3,103) = 22.81, *p* < 0.001, adjusted R^2^ = 0.38) in patients with PD (without APS).

### 3.3. Follow-Up after One Month

One month after discharge from the PD-MCT, 95 patients (83 PD, 12 APS) were reached and participated in a short telephone interview (21 patients were not reached). Among them, 22 (23.2%) were treated for 7–13 days (OPS8-97d.0), 71 (74.7%) patients for 14–20 days (OPS8-97d.1), and 2 (2.1%) patients for at least 21 days (OPS8-97d.2). Of note, 36.8% reported feeling better, 30.5% reported feeling unchanged, and 32.6% reported feeling worse after discharge from the hospital. In the univariate analyses, subjective well-being one month after discharge was related to improvement of the MDS-UPDRS III (*t* = 2.53; *p* = 0.014), younger age (*t* = 2.17; *p* = 0.034) and the absence of depression (chi-square test *p* = 0.038), but was not related to a shorter duration of the PD-MCT < 14 days (*p* = 0.802), sex (*p* = 0.727), NMSQ (*p* = 0.320), MoCA (*p* = 0.242), or the categorization into PD and APS (chi-square test *p* = 0.090).

In the logistic regression, subjective well-being one month after PD-MCT was associated with the absence of depression in patients with PD. The model was statistically significant (χ²(1) = 9.56, *p* = 0.008, Nagelkerke’s R² = 0.210). If patients were not depressed, they more likely reported feeling better after one month (OR = 4.690; 95% CI = 1.42–15.50; *p* = 0.011). 

### 3.4. Long-Term Dynamics of Health-Related Quality of Life

**Figure 4:**
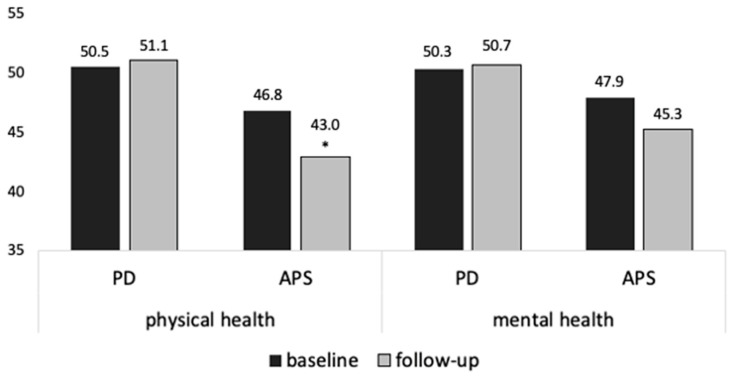
SF-12 summary scores of health-related quality of life at baseline and follow-up after 12 months (%). SF-12: Short-Form 12; *: *p* < 0.05.

The SF-12 questionnaire was conducted at baseline and after 12 months. At baseline, the SF-12 questionnaire was completed in 115 patients (99 PD, 16 APS). After one year, the SF-12 questionnaire was repeated in 84 patients (73 PD, 11 APS). Figure 4 shows the dynamics of the physical and mental health summary scores at baseline and 12 months after PD-MCT. The values were standardized according to the normative data of a representative German sample (50 ± 10) [9]. Higher values indicate a higher HR-QoL. At baseline, there were no significant differences between patients with PD and APS regarding their physical (*t* = 1.38; *p* = 0.171) or mental health summary scale (*t* = 0.90; *p* = 0.370). One year after discharge from PD-MCT, patients with APS reported more limitations regarding their physical health compared to patients with PD (*t* = −2.58; *p* = 0.012) and, therefore, a lower HR-QoL.

## 4. Discussion

In Germany, inpatient treatment of PD patients is often conducted as PD-MCT. It is a well-established and increasingly provided treatment option [3].

PD is a progressive disorder. It is assumed, that impaired motor function assessed by the MDS-UPDRS III score has a mean progression rate of around 5 points per year [10]. Our retrospective analysis suggests that PD-MCT may decelerate disease progression and has several beneficial treatment effects, with improvements in motor function assessed by the MDS-UPDRS III and motor performance assessed by the Tinetti test. This was demonstrated for both PD patients and patients with APS. From admission to discharge, a relative reduction in the MDS-UPDRS III score of nearly 30% could be achieved. This corresponds to an absolute reduction of −9.55 points, which is above the minimal clinically important difference for improvement of −3.25 points and is correspondingly clinically significant [11]. This is in line with earlier studies showing improvement of motor function after PD-MCT [4,5,6]. We found that improvement of MDS-UPDRS III after PD-MCT in patients with PD is more likely to occur in patients with higher MDS-UPDRS III at admission, lower age, and less depression. Accordingly, impaired motor function is an important predictor but not the only decisive factor for beneficial treatment. In addition to disease severity according to the MDS-UPDRS III, screening for and treatment of depression is essential. This is quite relevant because the prevalence of depressive symptoms and disorders in PD is high [12]. In our study population, 51.6% were identified to have depressive symptoms. This corresponds to the literature, with a mean prevalence of 54.3% in the inpatient setting [12].

As mentioned before, until now, data regarding the effectiveness of the shorter OPS8-97d.0 (7–13 days) have been missing. By means of these analyses, we were able to show that a shorter PD-MCT is also an effective treatment option for PD patients. Regarding motor function, there were no significant differences between patients who were treated for 7–13 days and patients who were treated for 14–20 days. However, this comparison must be made with caution, and we cannot make any confirmatory statement about the efficacy of short vs. long PD-MCT. The duration of PD-MCT may be influenced by selection bias. Physically less limited patients are more likely to be treated for a shorter period than more severely impaired patients. However, no differences regarding disease severity were found for either study cohort at baseline. To investigate whether shorter PD-MCT is as effective as longer PD-MCT, a randomized controlled trial is necessary.

Within our study, the prevalence of motor complications was high. At baseline, 137 patients (86.2%) reported symptoms assessed by the MDS-UPDRS IV. This prevalence is relatively high compared to frequency analyses, reporting levodopa-related dyskinesias with a median of 40% after four to six years of levodopa therapy [13]. Although it must be pointed out that the MDS-UPDRS IV is a frequently used scale for motor complications, it does not distinguish between different types of dyskinesias. Additionally, the prevalence of dyskinesia depends on the disease duration rather than the cumulative levodopa exposure [14]. However, the high frequency of motor complications has to be interpreted in line with the higher proportion of patients with the akinetic-rigid phenotype. These patients report dyskinesia more often than PD patients with a tremor-dominant phenotype [15]. Therefore, patients with an akinetic-rigid phenotype are more often transferred to PD-MCT [3], which is evident in our cohort.

Motor function is significantly improved by PD-MCT. Only three patients had no motor improvement. However, after one month, 32.6% of the patients reported feeling worse since their hospital stay. Although improvement of the MDS-UPDRS III increases the likelihood of subjective well-being after one month, not every patient who has objectively achieved motor improvement benefits subjectively from the therapy. A consideration of motor function alone does not adequately reflect the entirety of patient symptoms. In this regard, nonmotor symptoms seem to be of great importance. It was shown that subjective worsening after one month is related to depressive symptoms. If patients are not depressed, they have an increased likelihood of subjective well-being after one month. Therefore, screening and treatment for depression is essential, which is also reflected in the high prevalence of depression within our study cohort.

Motor and nonmotor symptoms have a huge impact on the quality of life of PD patients [16,17,18,19,20]. Additionally, there is evidence that PD-MCT might improve their quality of life [4]. Using the SF-12 questionnaire, we revealed that, one year after discharge from PD-MCT, patients with APS reported more limitations regarding their physical health than patients with PD. This may indicate a more beneficial effect of PD-MCT in patients with PD. However, it cannot be stated that this is a causal relationship. Rather, this should be seen in the context that patients with APS generally respond poorly to levodopa treatment, causing more pronounced symptoms compared to PD [21,22].

In Germany, the number of PD-MCTs is steadily increasing [3], especially for the longer duration of 14–20 days (OPS8-97d.1). Because the prevalence of PD is increasing, this is becoming a major challenge for the health care system. The crucial question is determining which patients can be treated as effectively or even more beneficially for a shorter period of 7–13 days (OPS8-97d.0). This is essential to manage the challenge to our health care system with limited capacities of highly specialized multidisciplinary PD inpatient treatment, as well to improve patient satisfaction. Therefore, a randomized controlled trial should be conducted to investigate the long-term effects of the different treatment durations and to identify patient characteristics that predispose to a beneficial treatment, especially within a shorter duration of 7–13 days.

Our study has some limitations that we need to point out. This was a retrospective analysis of patients treated at our neurology ward. The evaluation is based on single-center data. We did not perform a follow-up examination of motor function assessed by the MDS-UPDRS III because we used telephone calls for the follow-up. Additionally, not every patient was followed up, and the willingness to participate in the telephone interview was limited. We considered all patients who were treated within one year. Therefore, the data represent a heterogeneous population. The data revealed within our exploratory study are only descriptive and do not permit any statement on causal relationships between PD-MCT and the described changes during the follow-up assessments.

## 5. Conclusions

PD-MCT is an effective treatment option for inpatients with PD, leading to an improvement in motor and nonmotor symptoms. Within this study, we demonstrated that improvement of MDS-UPDRS III after PD-MCT in patients with PD was more likely to occur in patients with higher MDS-UPDRS III scores at admission, a younger age, and less depression. However, approximately one-third of the patients reported feeling worse one month after the hospital stay. A consideration of motor function alone does not adequately reflect the entirety of patient symptoms. Subjective worsening after one month is related to depressive symptoms. Therefore, screening for and treatment of depression seems to be essential. There is evidence that a shorter PD-MCT is an effective treatment option. However, a randomized controlled trial should be conducted to investigate the long-term effects of the different treatment durations and to identify patient characteristics that are predictive of a beneficial treatment within the shorter duration of 7–13 days. This would enable us to perform the increasingly requested PD-MCT more often and improve patient satisfaction.

## Figures and Tables

**Table 1:** Study population, baseline characteristics.

Variable	Value
Patients, *n*	159
PD	134 (84.3%)
APS	25 (15.7%)
Typ, *n*	
AR	108 (67.9%)
TD	13 (8.2%)
ND	35 (22.0%)
missing	3 (1.9%)
Age, y	72.5 ± 8.3
Sex, *n*	
female	59 (37.1%)
male	100 (62.9%)
Disease duration, y	9.4 ± 6.3
H&Y, median (IQR)	3.0 (2.5–4.0)
1.0	1 (0.6%)
1.5	11 (6.9%)
2.0	10 (6.3%)
2.5	30 (18.9%)
3.0	64 (40.3%)
4.0	33 (20.8%)
5.0	9 (5.7%)
missing	1 (0.6%)
MDS-UPDRS	73.7 ± 24.7
MDS-UPDRS Ia	4.0 ± 3.1
MDS-UPDRS Ib	10.6 ± 5.1
MDS-UPDRS II	19.7 ± 9.5
MDS-UPDRS III	34.2 ± 12.5
MDS-UPDRS IV	5.2 ± 3.7
LEDD, mg	796.4 ± 522.9
Tinetti test	18.4 ± 7.3
NMSQ	11.6 ± 5.1
BDI-II	12.5 ± 7.9
0–13, not depressed	72 (45.3%)
≥14, depressed	46 (28.9%)
missing	41 (25.8%)
HADS-D	8.0 ± 4.5
0–7, not depressed	55 (34.6%)
≥8, depressed	63 (39.6%)
missing	41 (25.8%)
Depression (BDI-II ≥ 14 or HADS-D ≥ 8)	
no	65 (40.9%)
yes	82 (51.6%)
missing	12 (7.5%)
MoCA	19.9 ± 4.7
≥26, normal	14 (8.8%)
21–25, mild cognitive impairment	61 (38.4%)
<21, dementia	79 (49.7%)
missing	5 (3.1%)

Values are given as the mean ± SD, unless otherwise indicated; categorial parameters are given as numbers and percentages. APS: Atypical Parkinsonian syndromes; AR: akinetic-rigid phenotype; BDI-II: Beck-Depression inventory II; HADS-D: Hospital Anxiety and Depression Scale; H&Y: Hoehn and Yahr stage; IQR: interquartile range; LEDD: levodopa equivalent daily dose; MDS-UPDRS: Movement Disorder Society sponsored revision of the unified Parkinson´s disease rating scale; MoCA: Montreal Cognitive Assessment; ND: not determined phenotype; NMSQ: nonmotor symptoms questionnaire; PD: Parkinson’s disease; TD: tremor-dominant phenotype.

## Data Availability

The data presented in this study are available on request from the corresponding author.
